# Supplementary material for: Comparative transcriptomics coupled to developmental grading via transgenic zebrafish reporter strains identifies conserved features in neutrophil maturation
Source: Nat Commun. 2024 Feb 27;15:1792. doi: 10.1038/s41467-024-45802-1 (PMC10899643; doi:10.1038/s41467-024-45802-1)
Supplement: Supplementary file 1 — Supplementary Information [file 41467_2024_45802_MOESM1_ESM.pdf]

Supplementary Information

Comparative transcriptomics coupled to developmental grading via transgenic zebrafish reporter strains identifies conserved features in neutrophil maturation

Stefanie Kirchberger,[1#*] Mohamed R. Shoeb,[1#] Daria Lazic,[1] Andrea Wenninger-Weinzierl,[1] Kristin Fischer,[1] Lisa E. Shaw,[2] Filomena Nogueira,[1, 3, 4], Fikret Rifatbegovic,[1] Eva Bozsaky,[1] Ruth Ladenstein, [1] Bernd Bodenmiller,[5, 6] Thomas Lion,[1,3,7] David Traver,[8] Matthias Farlik,[2] Christian Schöfer,[9] Sabine Taschner-Mandl,[1] Florian Halbritter[1§*] and Martin Distel[1§*]

[1]St. Anna Children's Cancer Research Institute (CCRI), Vienna, Austria; [2]Medical University of Vienna, Department of Dermatology, Vienna, Austria; [3]Labdia - Labordiagnostik GmbH, Vienna, Austria; [4]Medical University of Vienna, Center for Medical Biochemistry, Max Perutz Labs, Campus Vienna Biocenter, Vienna, Austria; [5]Department of Quantitative Biomedicine, University of Zurich, Zurich, Switzerland; [6]Institute of Molecular Health Sciences, ETH Zurich, Zürich, Switzerland; [7]Medical University of Vienna, Department of Pediatrics, Vienna, Austria; [8]Cell and Developmental Biology, University of California, San Diego, USA; [9]Medical University of Vienna, Division of Cell and Developmental Biology, Center for Anatomy and Cell Biology, Vienna, Austria;

# These authors contributed equally

§ These authors jointly supervised this work.

***Corresponding authors:**

stefanie.kirchberger@ccri.at; T +43 1 40470-4011 F +43 1 40470-7150

florian.halbritter@ccri.at; T +43 1 40470-4058, F +43 1 40470-7150

martin.distel@ccri.at; T +43 1 40470-4010, F +43 1 40470-7150

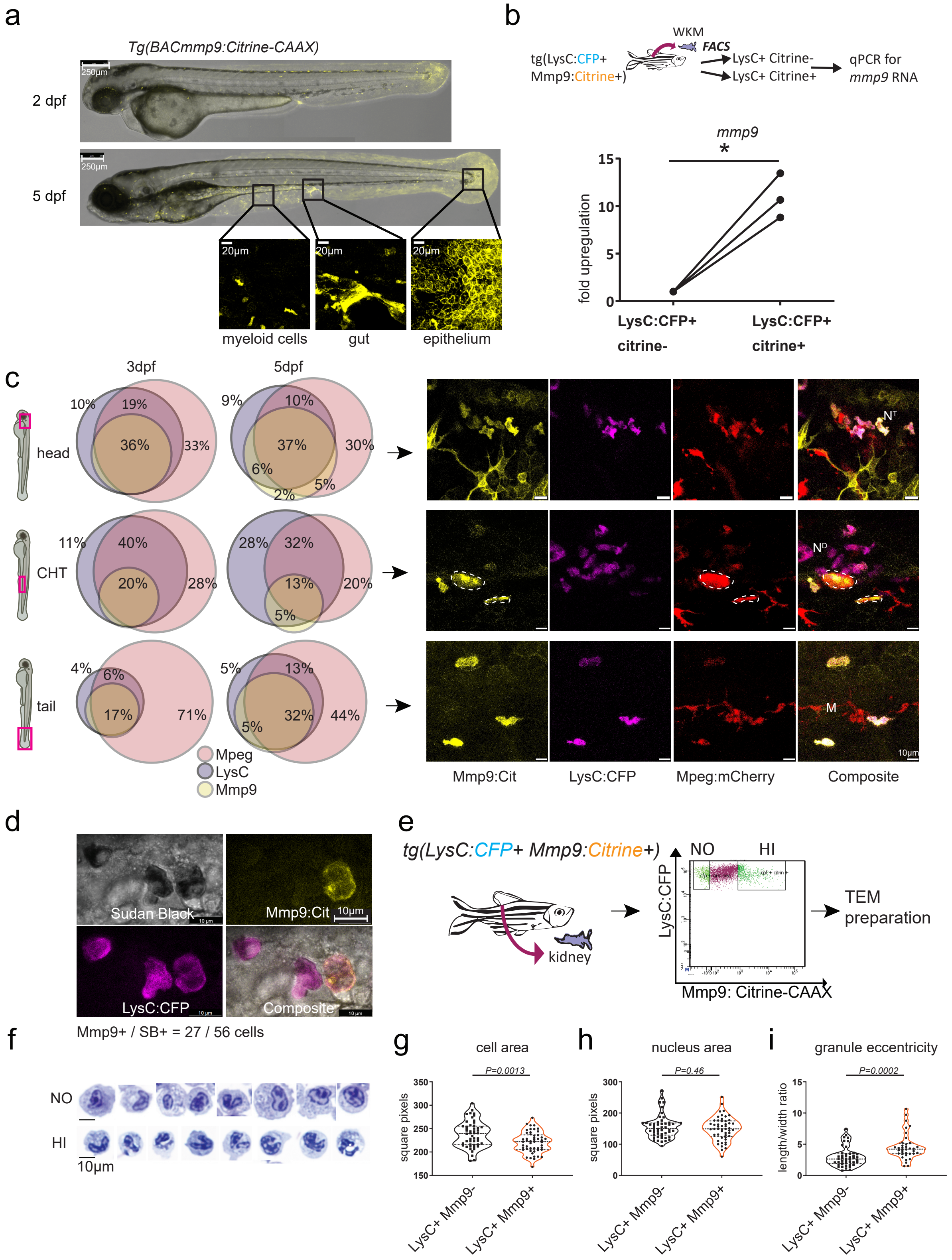
a
Tg(BACmmp9:Citrine-CAAX)
2 dpf
5 dpf
250µm
20µm
myeloid cells
gut
epithelium
b
tg(LysC:CFP+ Mmp9:Citrine+)
WKM
FACS
LysC+ Citrine-
LysC+ Citrine+
qPCR for mmp9 RNA
mmp9
*
fold upregulation
15
10
5
0
LysC:CFP+ citrine-
LysC:CFP+ citrine+
c
3dpf
5dpf
head
CHT
tail
10%
19%
36%
33%
9%
10%
37%
30%
6%
5%
2%
11%
40%
20%
28%
28%
32%
13%
20%
5%
4%
6%
17%
71%
5%
13%
32%
44%
5%
Mpeg
LysC
Mmp9
N^T
N^D
M
10µm
Mmp9:Cit
LysC:CFP
Mpeg:mCherry
Composite
d
Sudan Black
Mmp9:Cit
10µm
LysC:CFP
Composite
Mmp9+ / SB+ = 27 / 56 cells
e
tg(LysC:CFP+ Mmp9:Citrine+)
kidney
NO
HI
LysC:CFP
Mmp9: Citrine-CAAX
TEM preparation
f
NO
HI
10µm
g
cell area
P=0.0013
square pixels
350
300
250
200
150
h
nucleus area
P=0.46
square pixels
300
200
100
0
i
granule eccentricity
P=0.0002
length/width ratio
15
10
5
0
LysC+ Mmp9-
LysC+ Mmp9+

Supplementary Figure 1: Newly generated BAC transgenic line *Tg(BACmmp9:Citrine-CAAX)^{vi003}* reporting epithelial and leukocyte expression. a *Tg(BACmmp9:Citrine-CAAX)^{vi003}* zebrafish exhibit transcriptional activity of the *mmp9* locus in epithelial cells of the skin and gut and in dispersed cells. F1 *mmp9:Citrine* larvae were analyzed using fluorescence confocal microscopy on a Leica SP8 with a HCX PL APO CS 10x/0.40 DRY objective for Citrine (in yellow) at 2 and 5 dpf. To cover the whole larvae three individual pictures were taken and stitched. Scale bars 250 µm. Insets show expression in myeloid cells, the epithelia of the distal gut and the tail fin. **b** *Mmp9* RNA is enriched in whole kidney marrow cells from *Tg(lysC:CFP-NTR)^{vi002}/Tg(BACmmp9:Citrine-CAAX)^{vi003}* double transgenic adult fish sorted for Citrine expression. Whole kidney marrow (WKM) was isolated and FACS sorted into CFP+ Citrine- or CFP+ Citrine+ cells, which were analyzed by quantitative real-time PCR with primers for *mmp9*. n = 3, two-tailed paired t-test *P = 0.0144*. **c** Confocal analyses of co-localizations of LysC, Mmp9 and Mpeg in triple transgenic larvae *Tg(lysC:CFP-NTR)^{vi002} Tg(BACmmp9:Citrine-CAAX)^{vi003} Tg(mpeg:mCherry)^{gl23}* at 3 or 5 dpf in the head (n = 6 for 3 dpf; n = 5 for 5 dpf), CHT (caudal hematopoietic tissue) (n = 7 for 3 dpf; n = 3 for 5 dpf) or tail (n = 7 for 3 dpf; n = 3 for 5dpf) region. Venn diagrams showing average percentages (n = 3-7 larvae) and representative images at 5 dpf (right). Dashed lines indicate pigments. M, a typical Mpeg$^+$ macrophage; N^T, a LysC$^+$Mmp9$^+$Mpeg$^+$ neutrophil; N^D, a LysC$^+$Mmp9$^-$Mpeg$^+$ neutrophil. Acquired at a Leica TCS SP8 WLL microscope (HC PL APO CS2 40x/1.10 WATER objective). Scale bar = 10µm. **d** Sudan Black (SB) staining of *tg(lysC:CFP-NTR)^{vi002}/ Tg(BACmmp9:Citrine-CAAX)^{vi003}* larvae at 2 dpf. Mmp9$^+$ LysC$^+$ neutrophils demarcates a subpopulation of SB+ cells. **e** Preparation of FACS-sorted cells for TEM. **f** Toluidine blue-stained semi-thin sections of Mmp9$^-$ LysC$^+$ samples (NO = no expression; upper panel) display presence of immature cells indicated by significantly larger cell sizes **g** (two-tailed unpaired t-test *P=0.0013*), and unsegmented nuclear shapes whereas Mmp9$^+$ LysC$^+$ cells (HI = high expression; lower panel) show a differentiated morphology with a smaller cell volume and segmented nuclei. **h** Nuclear areas measured on stained semi-thin sections were similar (two-tailed unpaired t-test *P=0.46*). **i** Eccentricity of granules was measured in ImageJ on electron micrographs from Mmp9$^-$ LysC$^+$ (n = 5 cells; 44 granules) and Mmp9$^+$ LysC$^+$ (n = 2; 36 granules; two-tailed unpaired t-test *P=0.0002*).

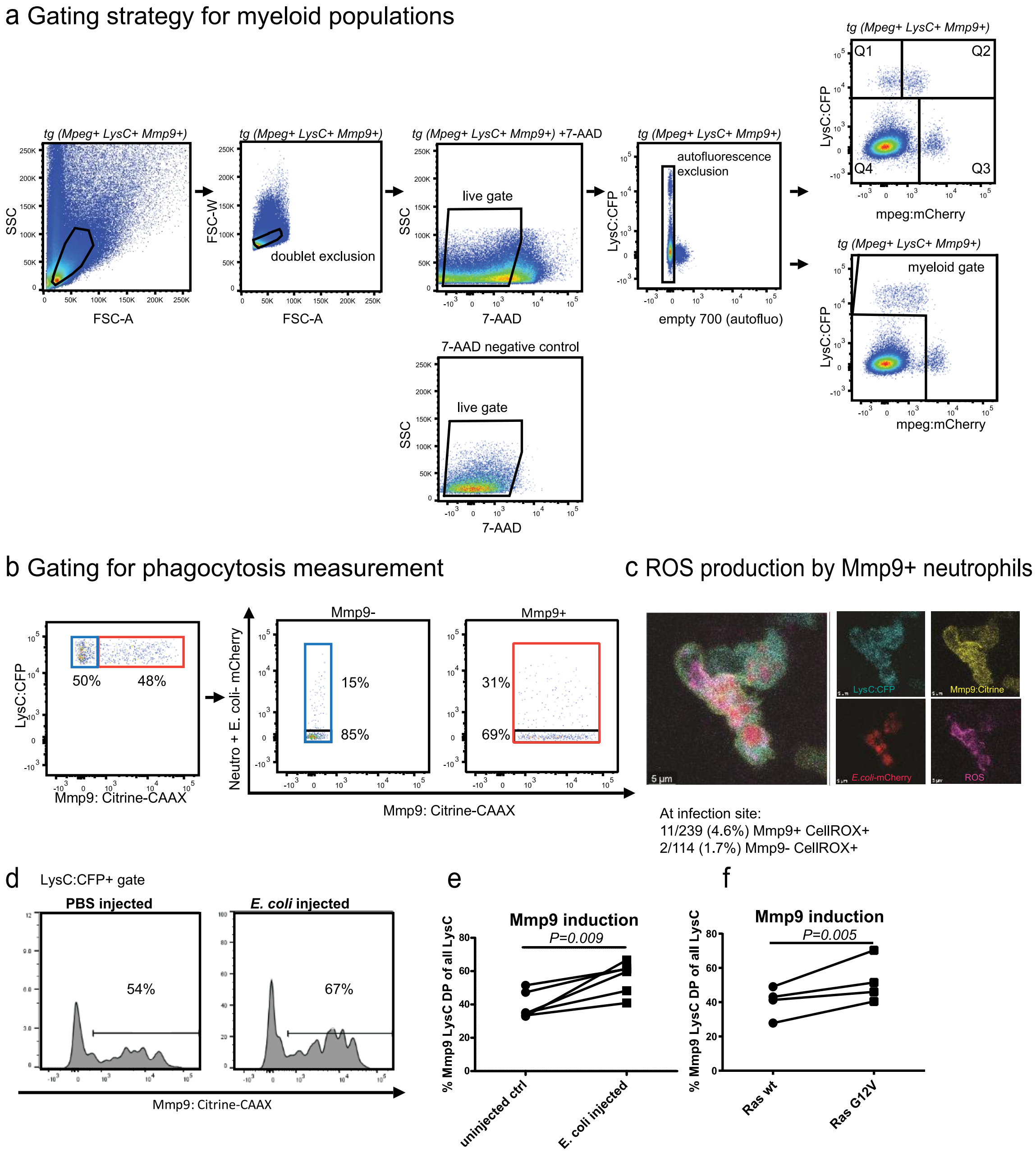

Supplementary Figure 2: Immune activation regulates frequency of mmp9+ neutrophils

a Gating strategy for myeloid populations as performed for Fig. 1c. **b** *In vivo* phagocytosis measurement. Gating strategy and representative flow cytometry plots of cells isolated from *E. coli* injected larvae 6 hpi, pre-gated on *lysC:CFP*$^+$ cells (left) and further gated on Mmp9$^-$ (middle panel) and Mmp9$^+$ (right panel) populations, showing a higher percentage of Mmp9$^+$ cells with bacterial cargo. **c** Reactive oxygen species (ROS) production by Mmp9$^+$ LysC$^+$ neutrophils. Visualization of CellROX Deep red after otic vesicle injection of *E.coli*-mCherry at 90 minutes post injection. Imaged on a Leica SP8 with a HC PL APO CS2 63x/1.40 OIL. **d** Representative flow cytometry histograms showing induction of *mmp9:Citrine* expression in neutrophils gated on *lysC:CFP*$^+$ by wounding through PBS injection (left) or administration of bacteria (right). **e** Graph summarizing results of six independent activation experiments (~20 larvae each) analyzed by flow cytometry indicating an increased

frequency of Mmp9$^+$ cells within the *lysC:CFP$^+$* neutrophil population upon *E. coli* injection (6 hpi). Paired t-test; *P = 0.009.* **f** Upregulation of *mmp9:Citrine* in neutrophils gated on *lysC:CFP$^+$* in the kita pre-neoplastic melanoma model *Et(kita:GAL4)^{hzm1}x Tg(HRAS_G12V:UAS:CFP)^{vi004}* at 3 dpf. n=4. Paired t-test; *P = 0.005.*

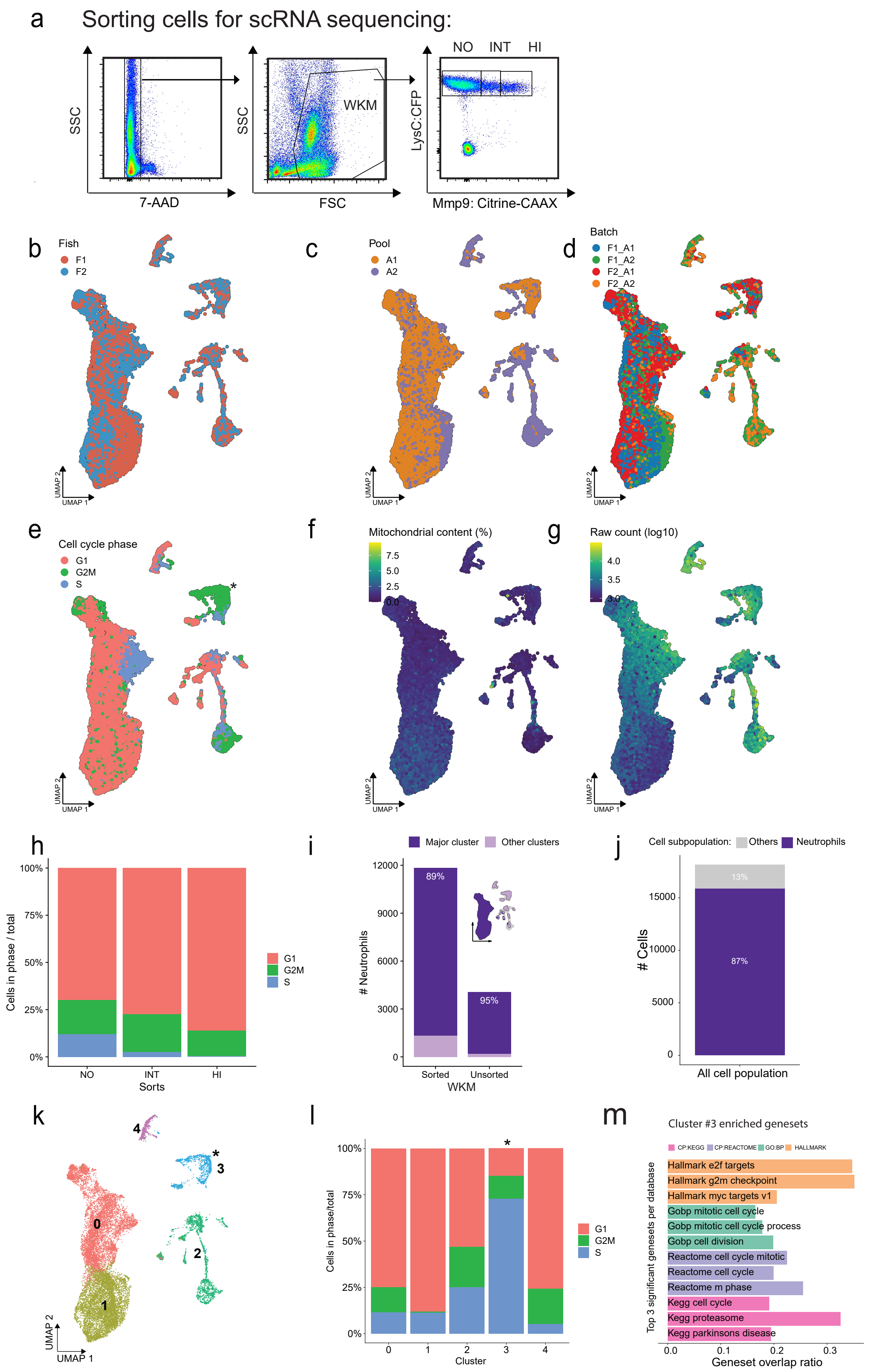

a
Sorting cells for scRNA sequencing:
SSC
7-AAD
FSC
WKM
LysC:CFP
Mmp9: Citrine-CAAX
NO
INT
HI
b
Fish
F1
F2
c
Pool
A1
A2
d
Batch
F1_A1
F1_A2
F2_A1
F2_A2
e
Cell cycle phase
G1
G2M
S
f
Mitochondrial content (%)
g
Raw count (log10)
UMAP 1
UMAP 2
h
Cells in phase / total
Sorts
NO
INT
HI
i
Major cluster
Other clusters
Neutrophils
89%
95%
Sorted
Unsorted
WKM
j
Cell subpopulation:
Others
Neutrophils
Cells
13%
87%
All cell population
k
l
Cells in phase/total
Cluster
m
Cluster #3 enriched genesets
CP:KEGG
CP:REACTOME
GO:BP
HALLMARK
Top 3 significant genesets per database
Hallmark e2f targets
Hallmark g2m checkpoint
Hallmark myc targets v1
Gobp mitotic cell cycle
Gobp mitotic cell cycle process
Gobp cell division
Reactome cell cycle mitotic
Reactome cell cycle
Reactome m phase
Kegg cell cycle
Kegg proteasome
Kegg parkinsons disease
Geneset overlap ratio

Supplementary Figure 3: Workflow and quality control of scRNA-seq of zebrafish kidney marrow neutrophils

a FACS gating strategy for neutrophil isolation for 10x Genomics analysis. Whole kidney marrow (WKM) cells were sorted on a FACS Aria II gating on live (7-AAD negative), Forward scatter (FSC)/ side scatter (SSC) gate to exclude debris and dead cells. Neutrophils were gated on *lys:CFP+* and different levels of *Mmp9:Citrine-CAAX* expression. UMAP (Uniform Manifold Approximation and Projection) representation of WKM and sorted neutrophils labelled by biological (**b**, Fish) and technical covariates (**c**, Pool = FACS well; **d,** Batch), inferred cell cycle phase (**e**), proportion of mitochondrial reads (**f**), and $\log_{10}$ raw UMI counts (**g**). **h** Bar plot showing the proportion of each cell cycle phase per sorted subset. NO = no expression; INT = intermediate expression; HI= high expression. **i** Stacked bar plot showing the number of neutrophils in the sorted and unsorted WKM subsets, along with the relative frequency of cells from neutrophil-major and neutrophil minor clusters displayed on a small UMAP in the same plot. **j** Stacked bar plot showing the total number and relative frequency of neutrophils. **k** UMAP of single-cell RNA-seq data (n = 18,150 cells) showing Seurat clustering results using resolution *0.05*. The cycling cell cluster is labelled with an asterisk (*). **l** Stacked bar plot showing the percentage of cells per cell-cycle phase in each cluster. The cycling cell cluster is labelled with an asterisk (*). **m** Top 3-enriched gene sets per database (ranked by FDR) from hypeR overrepresentation analysis of cluster 3 markers.

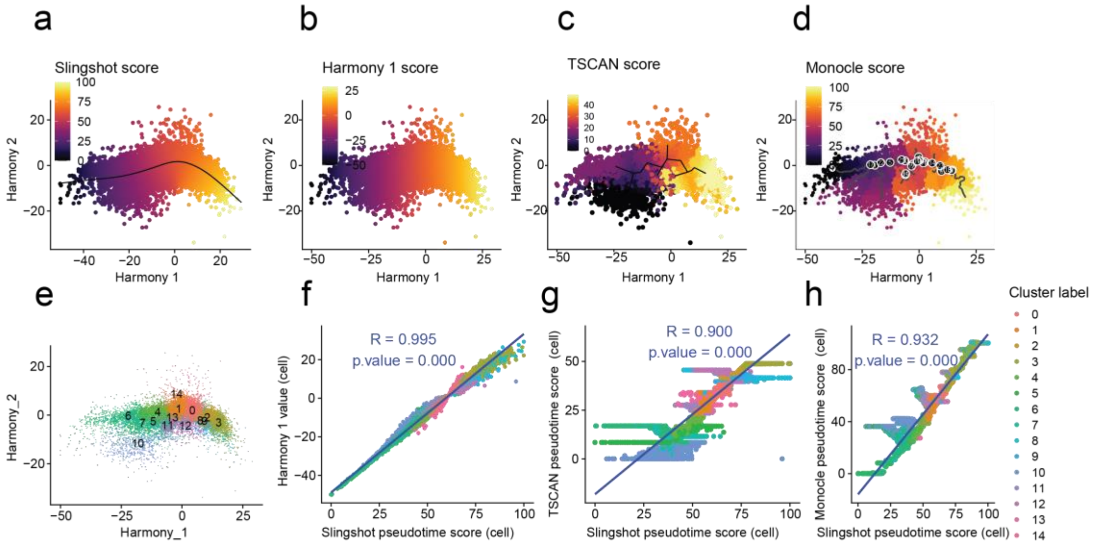

Supplementary Figure 4: Comparison of different trajectory inference methods to Slingshot. The top panels show the trajectories and pseudotime scores returned by Slingshot (**a**), “Harmony 1” (**b**), TSCAN (**c**), and Monocle (**d**). The bottom row shows the clusters (1-14) (**e**) used for tools that required pre-defined clusters (TSCAN, Monocle) and scatterplots comparing Slingshot pseudotime (x-axis) with the pseudotime scores returned by “Harmony 1”(**f**), TSCAN (**g**), and Monocle (**h**) on the y-axis.

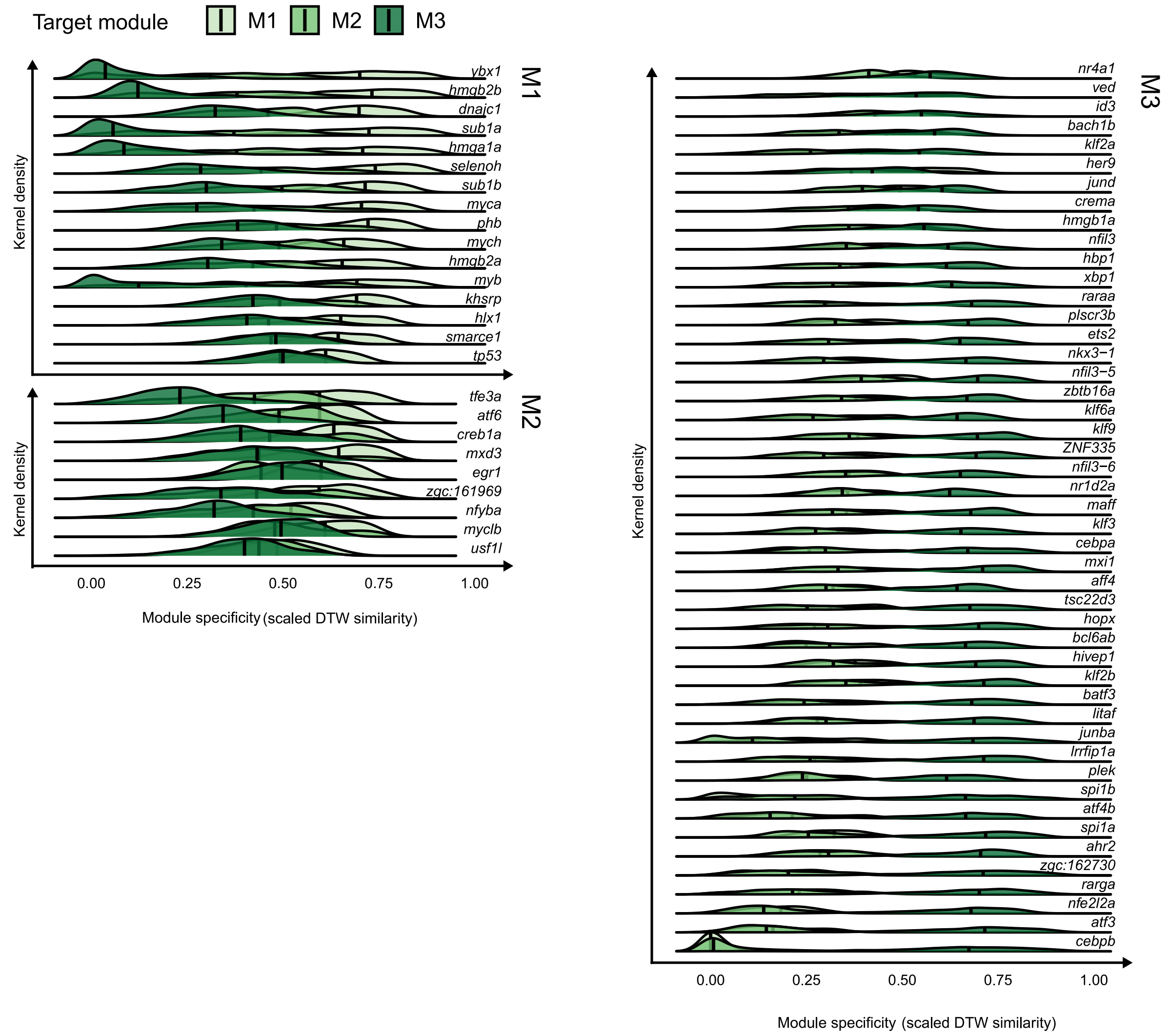

Supplementary Figure 5: Identification of candidate regulators per gene module by dynamic time warping analysis

Ridge plots showing the distribution of transcription factor specificity for each gene across the three modules (M1-3). Y-axis show all transcription factors grouped by the corresponding module. The X-axis indicates the specificity score of a transcription factor with respect to the genes of each module. Briefly, specificity measures the similarity of a target gene expression pattern to a candidate regulator, compared to all other transcription factors (see *Methods* for the formula).

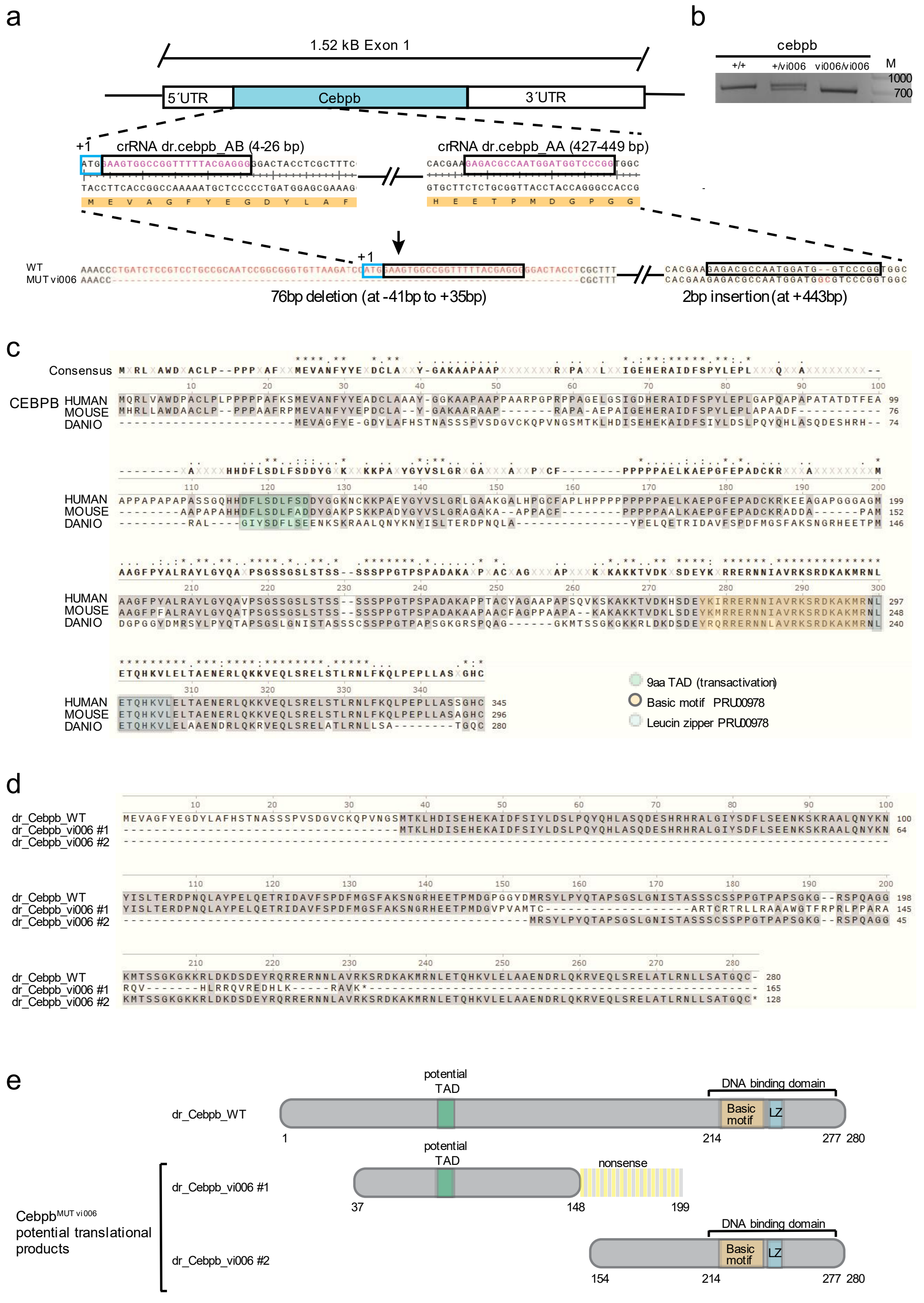

Supplementary Figure 6: Generation of Cebpb^{vi006} mutant by CRISPR-Cas9

a Genetic locus scheme of the single-exon gene *cebpb* in zebrafish. Black boxes indicate the two crRNA sequences used in combination for targeting *cebpb*. The *cebpb* mutant line *vi006* was generated by microinjecting Cas9-RNPs into fertilized *Tg(lysC:CFP-NTR)^{vi002}/Tg(BACmmp9:Citrine-CAAX)^{vi003}* eggs. *cebpb$^{MUT\ vi006}$* contains a 76 bp deletion (-41 to +35 bp) and a 2 bp insertion (at +443 bp). **b** *cebpb* mutant genotyping by PCR. Agarose gel showing *cebpb^{WT}* (787bp), *cebpb$^{WT/MUT\ vi006}$* (787/713 bp) heterozygous and *cebpb$^{MUT\ vi006/MUT\ vi006}$* (713 bp) homozygous bands. **c** Alignment of human CEBPB (P17676), mouse CEBPB (P28033) and zebrafish

Cebpb (ENSDARP00000062701) amino acid sequences by Clustal Omega indicates a conserved domain architecture including a potential transactivation domain (TAD) and a bZIP motif. **d** Alignment of wildtype and mutant Cebpb amino acid sequences. Zebrafish C/ebp-β is highly homologous to mouse and human C/EBP-β in the TAD and bZip domains In mammals the single exon gene C/EBP-β can produce three N-terminally truncated isoforms translated from a single RNA containing successive in-frame start codons[A1]. The shortest truncated isoform LIP lacks the transactivation domain (TAD) and is considered a dominant repressor by binding and functionally neutralizing dimerization partners of the C/EBP, JUN and ATF families[A2, A3]. The zebrafish *cebpb*$^{MUT\ vi006}$ line could potentially lead to two translational products: #1; an N-terminally shortened form by 36 aa containing the TAD domain but no DNA-binding domain; #2 an N-terminally shortened form by 153 aa containing no TAD, but a DNA-binding domain, similar to mammalian LIP. **e** Domain structure of potential *cebpb*$^{MUT\ vi006}$ products. LZ = leucine zipper.

Fig. 7a.Cross-correlation lags of selected genes relative to zebrafish

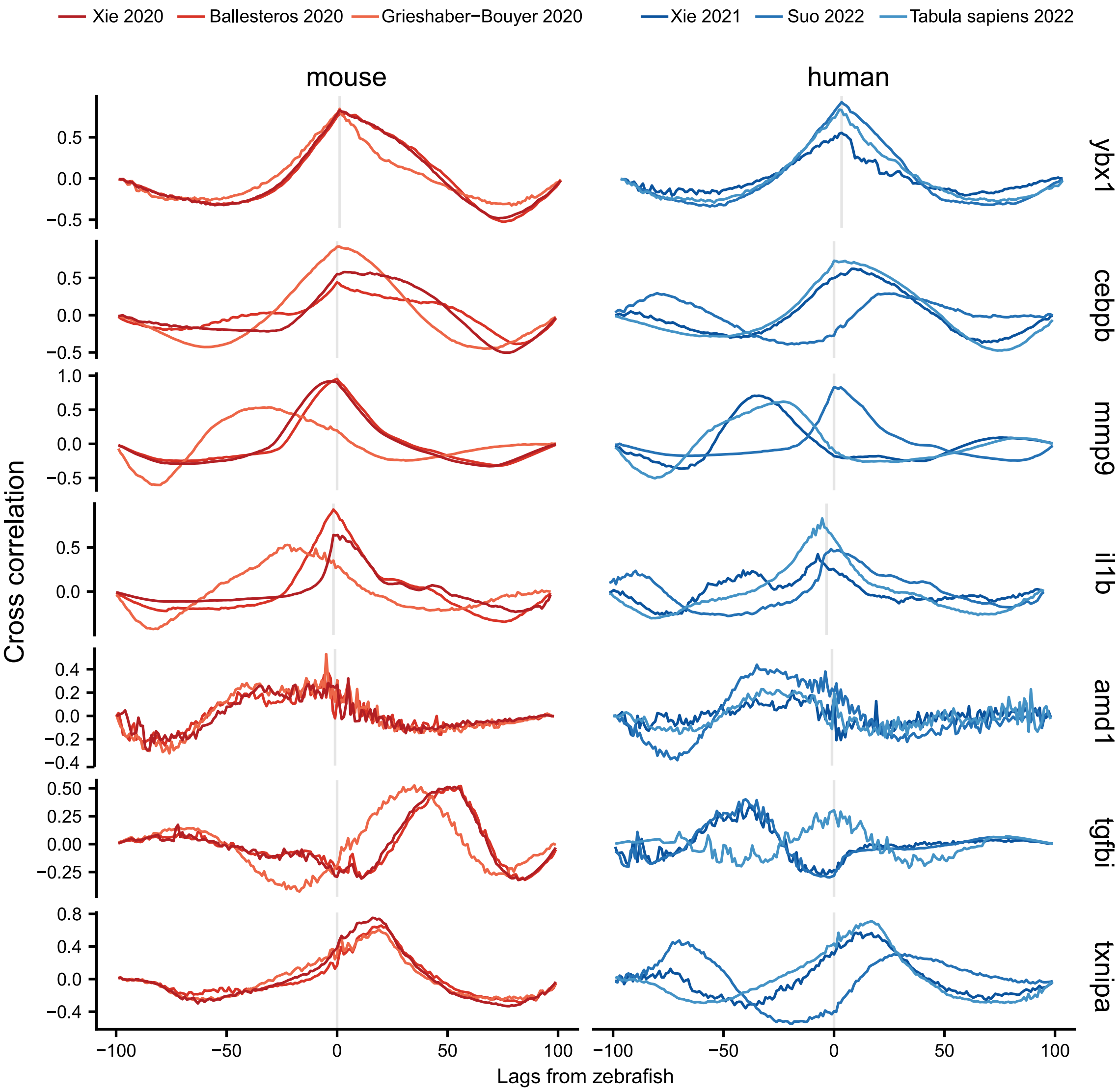

Fig 7b.Cross-correlation lags from zebrafish reference for all common differentially expressed genes (M1).

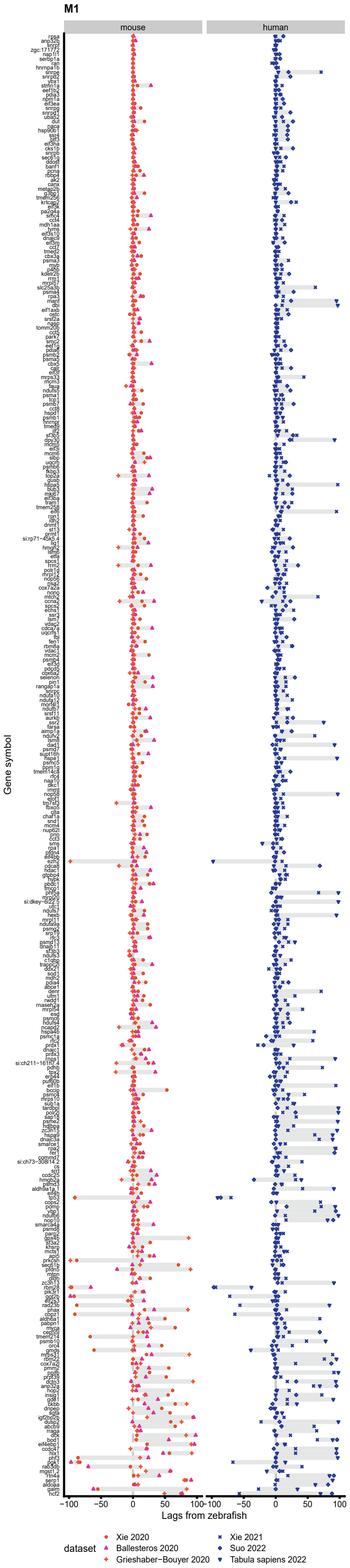

Fig. 7b.Cross-correlation lags from zebrafish reference for all common differentially expressed genes (M2)

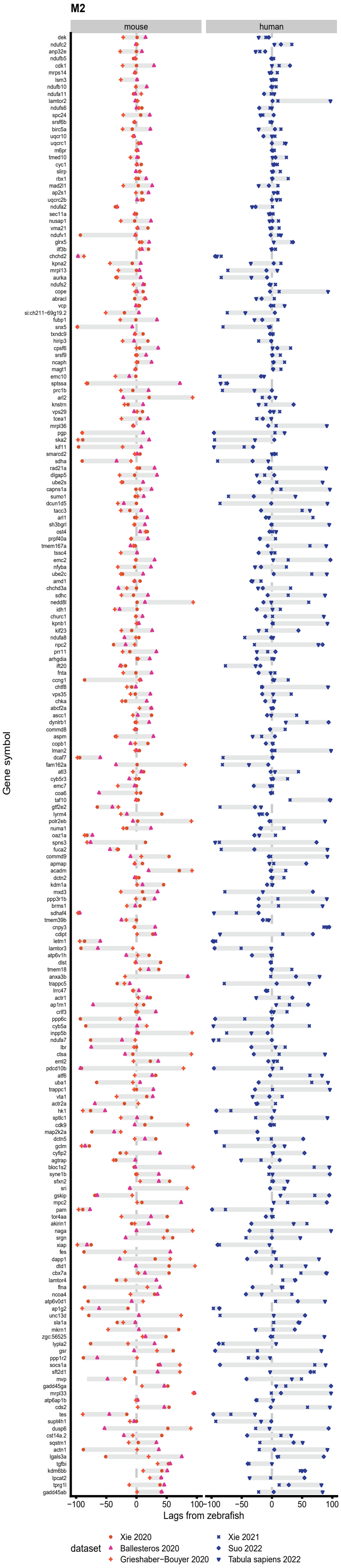

Fig 7b.Cross-correlation lags from zebrafish reference for all common differentially expressed genes (M3)

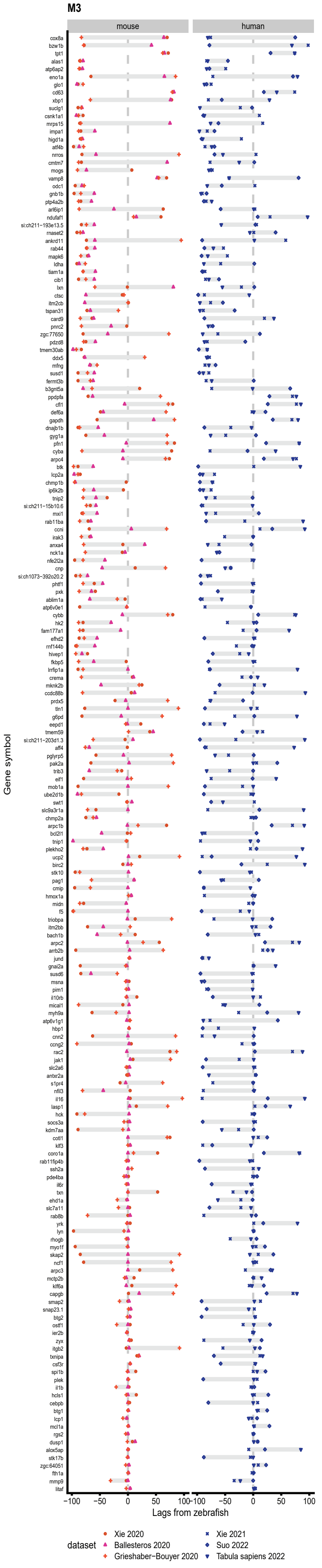

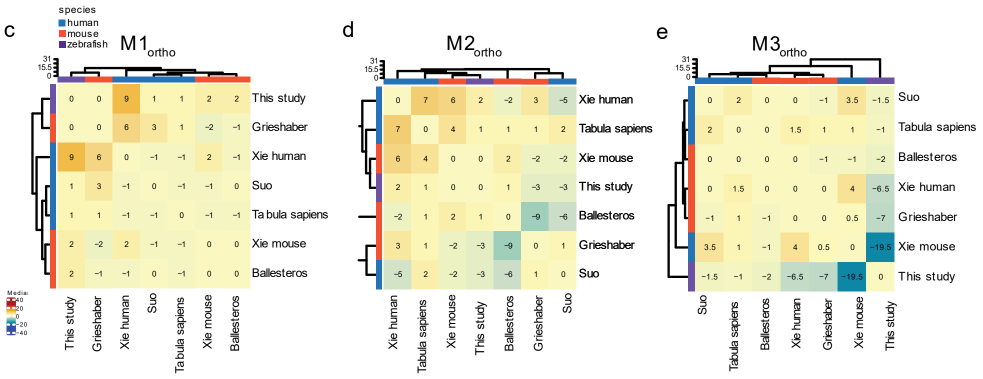

Supplementary Figure 7: Cross-correlation analysis of gene expression during zebrafish and mammalian neutrophil maturation a Line plots showing seven selected examples of genes with different cross correlation patterns. *ybx1* shows a good concordance across species with highest correlation centered at zero lags from zebrafish. *mmp9* expression shows a noticeable lag to an earlier maturation phase relative to zebrafish with cross correlation peaking at negative lags. *tgfbi* shows contradictory pattern between mouse and human. **b** Cross-correlation lags for all commonly differentially expressed genes in all datasets. A positive value indicates a gene is expressed at a later stage relative to its expression stage in zebrafish, and *vice versa*. **(c-e)** Heatmaps displaying the median co-phenetic distance of orthologue expression between the indicated datasets, ordered by hierarchical clustering with complete linkage.

a. HAY signature genes included in the zebrafish signature

HAY _zfish_common	zfish_module
ctnnb1	$M1_{pan}$
gpx4b	
ifi30	
mhc1uba	
pfdn5	
uba5 2	
dusp6	$M2_{pan}$
lgals3a	
sec 11a	
sgk1	
atp6v1 g1	$M3_{pan}$
b3gnt5a	
birc2	
cebpb	
crip1	
ctss2.1	
hbegfa	
hk2	
hmox1a	
hpse	
il1b	
irf2bp2b	
itm2ba	
lcp2a	
lgals2a	
mapk6	
metrnla	
nfkb iaa	
notch2	
ptp4a 2b	
rhoab	
sus d6	
txnipa	
zgc:158 343	

b. Application of HAY_immature signature on Ramirez et al . in-vitro differentiated neutrophils

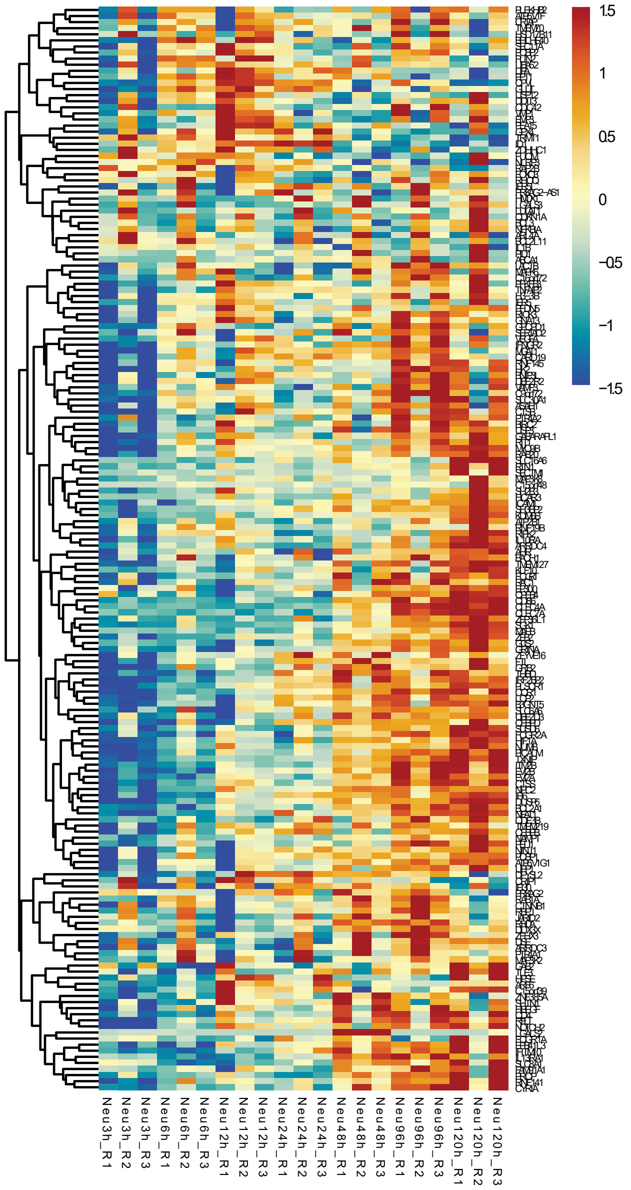

Supplementary Figure 8: **HAY_immature signature pre-dominantly includes late neutrophil maturation genes**. **a** Evaluation of the overlap between zebrafish gene modules and genes from HAY_immature signature[55]. **b** Heatmap analysis of HAY_immature signature on Ramirez *et al*.[51] *in vitro* differentiated neutrophils.

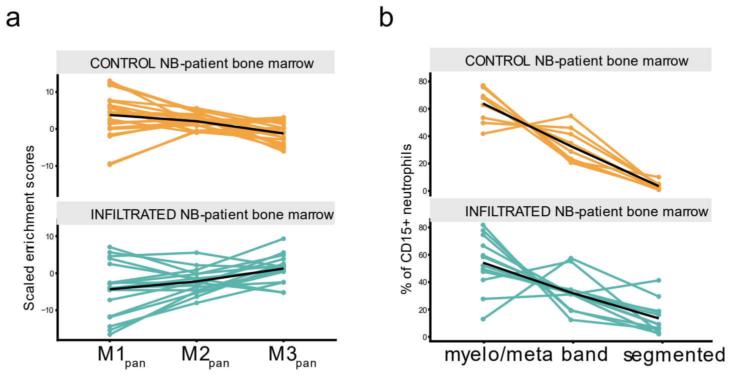

Supplementary Figure 9: Human Neuroblastoma-BM RNA-seq analysis. a Scaled ssGSEA[57] enrichment scores for $M1_{pan}$, $M2_{pan}$, and $M3_{pan}$ genes in bulk RNA-seq data[56] from 38 bone marrows of patients with metastatic (n = 17, infiltrated) and localized (n = 21, control) neuroblastoma (NB). Line plots show scores for each dataset. **b** Frequency of myelocytes and metamyelocytes, band cells and segmented neutrophils among all $CD15^+$ neutrophils in BM cytospin samples from patients with metastatic (n = 12, infiltrated) and localized (n = 9, control) neuroblastoma. Samples were stained with Iridium and anti-CD15-Bi209, analyzed by IMC and assessed for their morphology in QuPath. Line plots show frequencies of each neutrophil subtype per patient.

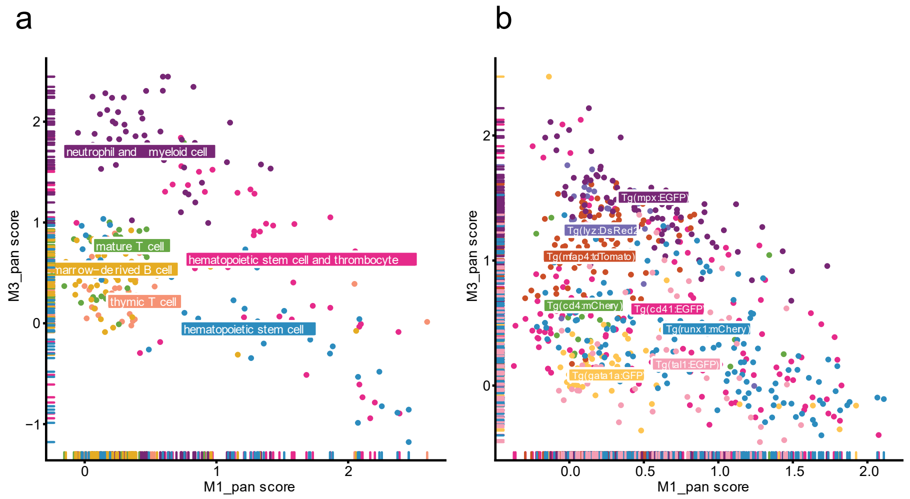

Supplementary Figure 10: Specificity of neutrophil maturation signatures in zebrafish hematopoietic reference data. Scatterplots comparing $M1_{pan}$ and $M3_{pan}$ signature scores in different hematopoietic cell types from Athanasiadis *et al.* 2017 (**a**) and Tang *et al.* 2017 (**b**)[38,24]. Module scores were calculated by *AddModuleScore* function from Seurat package. In both cases, we found that mature "neutrophils and myeloid cells" and cells with neutrophil-linked reporters *lyz* and *mpx* exhibited the strongest M3_pan scores while having very low M1_pan scores. On the contrary, subsets of HSPCs and/or *runx1* progenitors had a high M1_pan score and low M3_pan score. Both datasets also showed (few) neutrophils with medium scores for both signatures, which might represent cells at intermediate maturation stages. Cells of other hematopoietic lineages had a low score for both M1_pan and M3_pan (e.g., cd4 / T cells).

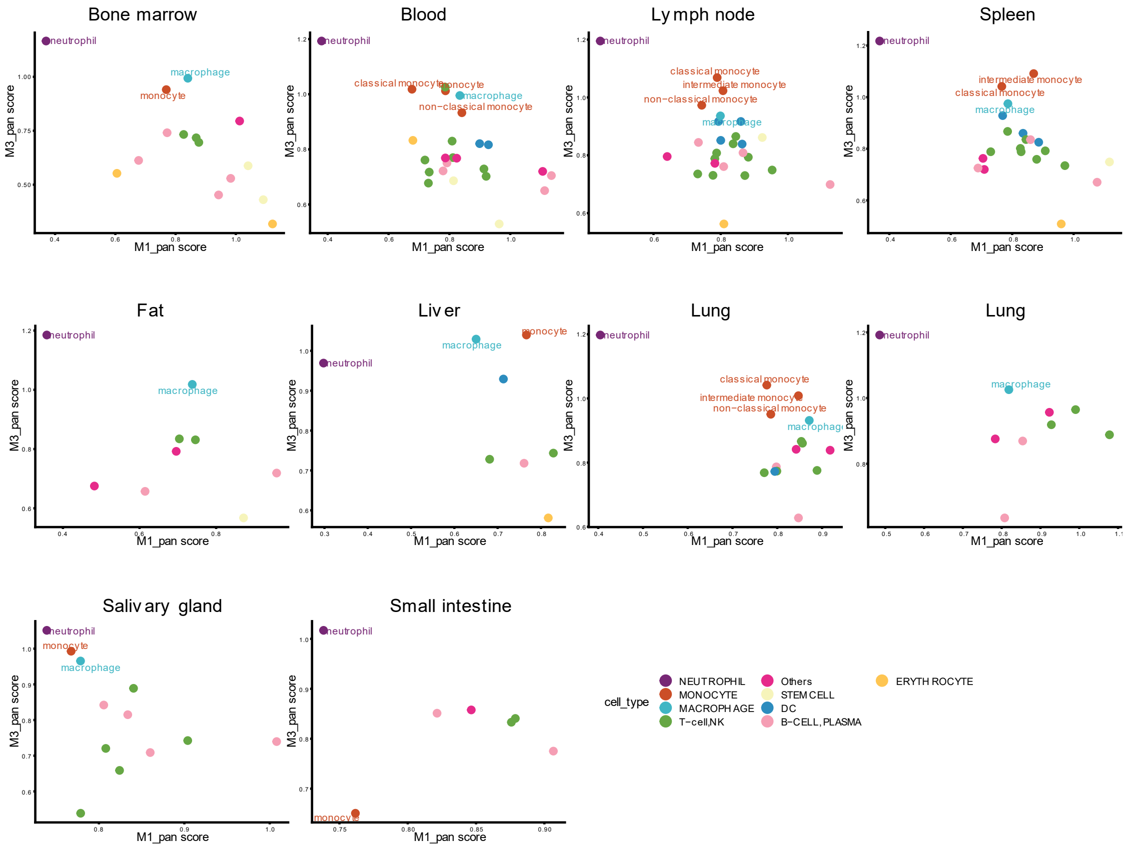

Supplementary Figure 11: Specificity of neutrophil maturation signatures in Human Cell Atlas data. Scatterplots comparing $M1_{pan}$ and $M3_{pan}$ signature scores across pseudo-bulk data of human [53]hematopoeitic cell types from Tabula Sapiens Consortium 2022 across different tissues. Module scores were calculated by *gsva* function from GSVA package. The two module scores clearly separate neutrophils from the other cell types across all tissues.

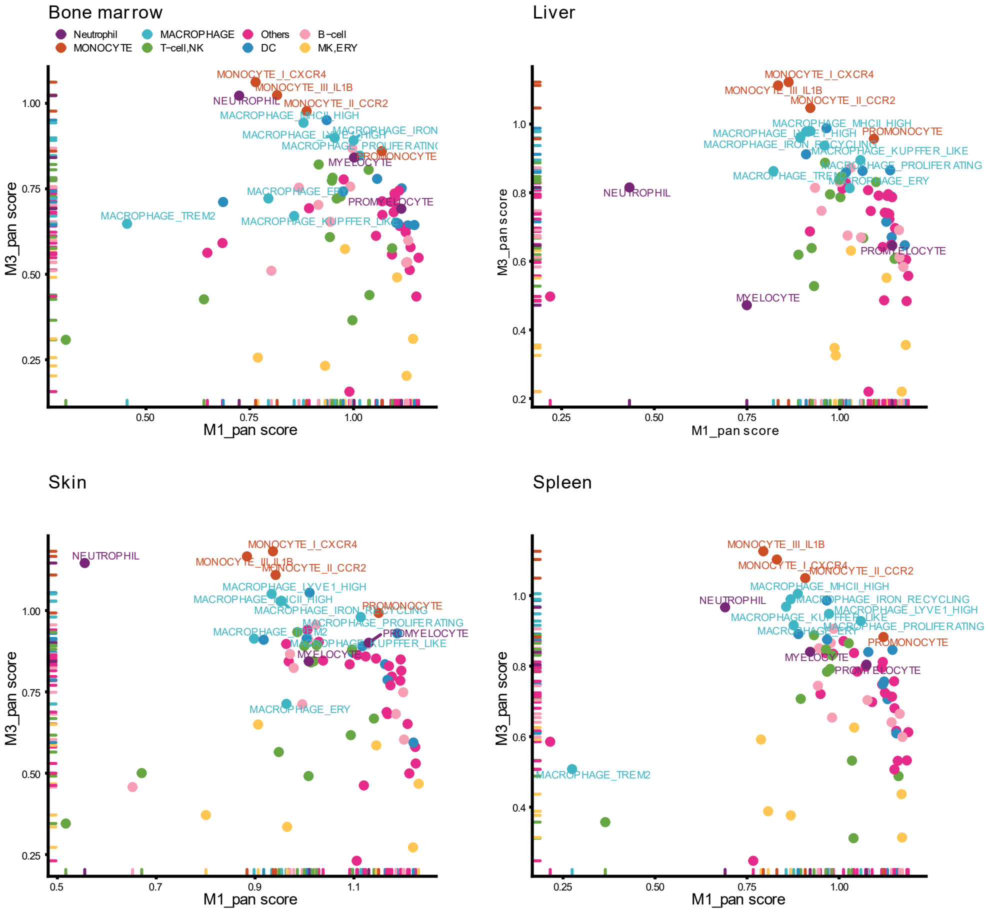

Supplementary Figure 12: Specificity of neutrophil maturation signatures in human development atlas. Scatterplots comparing $M1_{pan}$ and $M3_{pan}$ signature scores across pseudo-bulks of human hematopoietic maturation stages from Suo 2022 *et al.* across different tissues[52]. Module scores were calculated by *gsva* function from GSVA package. The mature neutrophil stage (NEUTROPHIL) shows high M3_pan score and low M1_pan score, the immature stage (PROMYELOCYTE) shows the opposite pattern, and the intermediate stage (MYELOCYTE) is in-between. In combination, the scores from the two modules resolve and separate the three neutrophil stages across tissues achieving the correct order of maturation (albeit individual scores might be higher, e.g., M3_pan for some monocytes).

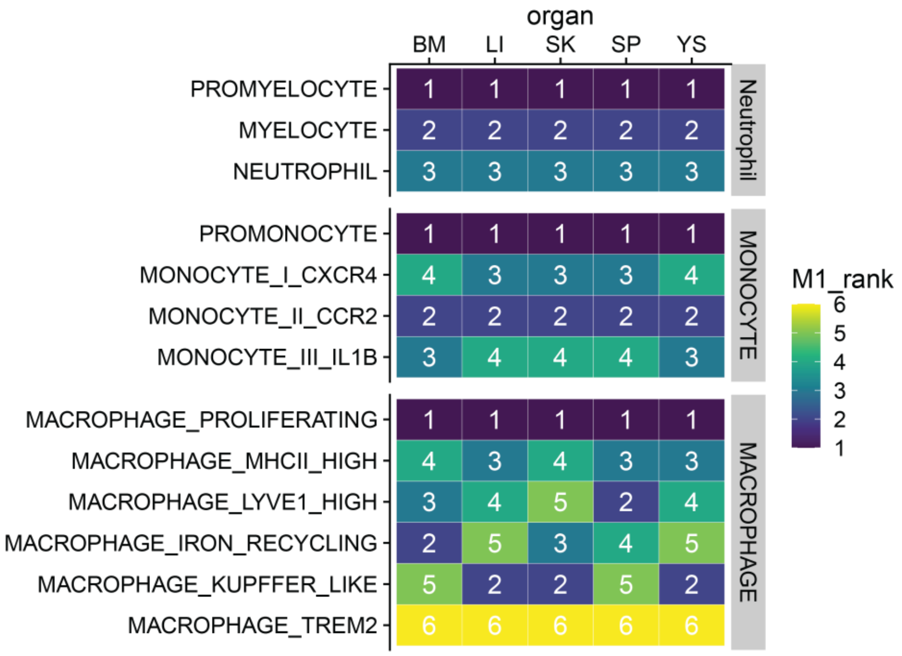

Supplementary Figure 13: Order of maturation stages of myeloid cells based on M1$_{pan}$ score. Heatmap showing the order maturation stages of each cell type across different tissues. The numbers are the ranks where lower values indicate earlier maturation stage. X-axis shows the tissues and y-axis shows maturation stages ordered following the original publication Suo *et al.* 2022. Neutrophils are the only cell type where the correct order is attained and maintained across tissues.

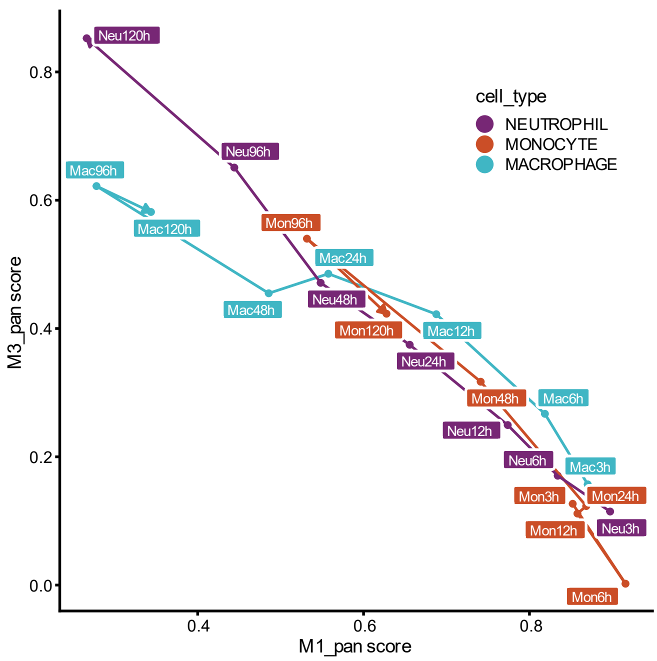

Supplementary Figure 14: Specificity of neutrophil maturation signatures in human in-vitro data. Scatterplot comparing $M1_{pan}$ and $M3_{pan}$ signature scores in differentiating human myeloid cell types from Ramirez *et al*. 2017. Module scores were calculated by *gsva* function from GSVA package. The results of this dataset show that differentiating neutrophils (Neu) at early time points had higher M1_pan and lower M3_pan scores compared to monocytes (Mon) and macrophages (Mac), which gradually switches to the opposite pattern at late differentiation timepoints. Additionally, while the neutrophil maturation trajectory is correctly recapitulated based on the two modules scores, they completely fail to order the monocyte maturation time points and incorrectly reverse the order of 120 and 96 hours in macrophages.

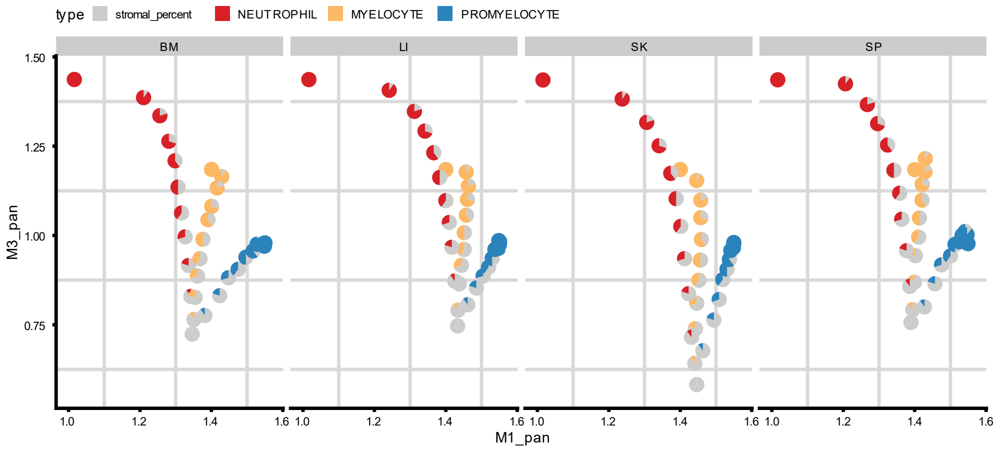

Supplementary Figure 15: Computational mixing of neutrophil maturation stages and stromal cells. Scatter pie plot of $M1_{pan}$ and $M3_{pan}$ signature scores across a gradient of neutrophil and stromal cells mixes from different tissues from Suo *et al.* 2022. Module scores were calculated by *gsva* function from GSVA package. The mixing process was repeated 20 times, and the median scores are shown in the figure. The analyses shown here evaluate how well maturation status could be assessed in the context of different surrounding tissues. We performed an *in silico* titration experiment in which we mixed in neutrophils at different maturation stages (promyelocyte, myelocyte, neutrophil) and at different ratios (0%, 10%,…, 100%) with stromal cells from different tissues to create pseudo-bulk samples with known neutrophil contributions. The results show how the modules scores' order and resolve the mixtures into increasingly different groups with increased neutrophil. The distinction between stages can be made at neutrophil percentages as low as 20%.

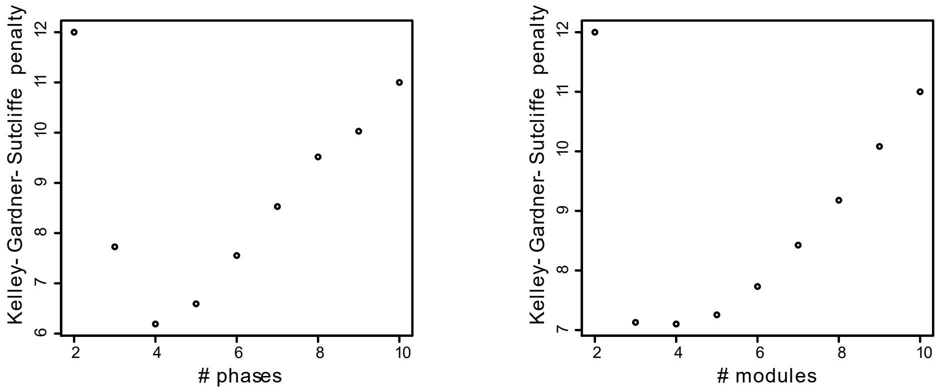

Supplementary Figure 16: Optimal selection of the number of phases and modules. Using Kelley-Gardner-Sutcliffe penalty for optimal pruning of the hierarchical cluster tree**.** The minimum penalty corresponds to the suggested number of clusters.

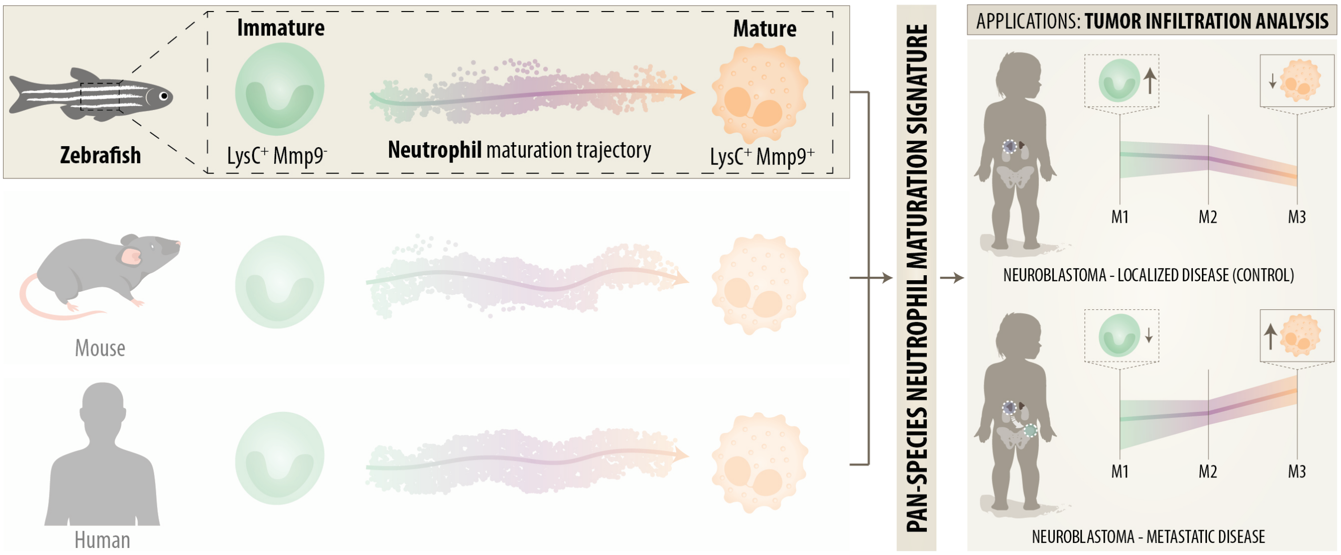

Supplementary Figure 17: Graphical abstract

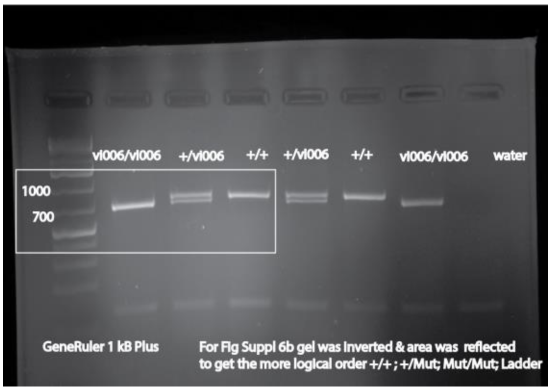

Supplementary Figure 18: Uncropped scan of the gel shown in Supplementary Figure 6b. The gel was inverted and the area was reflected to get the more logical order +/+; +/vi006; vi006/vi006. GeneRuler 1kB Plus was used as ladder.

Supplementary Table 1:

Primers used for qPCR

cebpb	CAAAAGTAACGGGCGACAC	CATTTTACCGCCCGCTTGAG
ef1a	CTACCCTCCTCTTGGTCGCT	GGAACGGTGTGATTGAGGGAA
fcer1gl	TGGAGACCTCTATCAGGATCTGG	AATGTGGGACATGGTTACAGC
il1b	AAAGTGCGCTTCAGCATGTC	ACCCGCTGATCTCCTTGAGT
lyz	GCCTACTGGGAAAGCAGGTT	AAGCAAGTCTGAACAGGCCA
mcl1a	AACAAGAGCTGGCATGGGTT	CTTCTAAGCGCCTCGGTGAG
mmp9	GCTTCTGTCCCAGTGAGCTT	GCCGTATCTCTGTTAGGGCA
spi1b	AGAGAGGGTAACCTGGACTGG	TAATCCCAAGAGTGATCGTTCTGA

Additional References in Supplementary Information

A1. Descombes, P. & Schibler, U. A liver-enriched transcriptional activator protein, LAP, and a transcriptional inhibitory protein, LIP, are translated from the same mRNA. *Cell* **67**, 569-579 (1991).

A2. Hsu, W., Kerppola, T.K., Chen, P.L., Curran, T. & Chen-Kiang, S. Fos and Jun repress transcription activation by NF-IL6 through association at the basic zipper region. *Mol Cell Biol* **14**, 268-276 (1994).

A3. Vallejo, M., Ron, D., Miller, C.P. & Habener, J.F. C/ATF, a member of the activating transcription factor family of DNA-binding proteins, dimerizes with CAAT/enhancer-binding proteins and directs their binding to cAMP response elements. *Proc Natl Acad Sci U S A* **90**, 4679-4683 (1993).
