# Supplementary material for: Comparative transcriptomics coupled to developmental grading via transgenic zebrafish reporter strains identifies conserved features in neutrophil maturation
Source: Nat Commun. 2024 Feb 27;15:1792. doi: 10.1038/s41467-024-45802-1 (PMC10899643; doi:10.1038/s41467-024-45802-1)
Supplement: Supplementary file 3 — Description of Additional Supplementary Files [file 41467_2024_45802_MOESM3_ESM.pdf]

Description of Additional Supplementary Files

File Name: Supplementary Data 1
Description: Maturation-associated genes differential expression statistics using Slingshot, modules membership, and signatures assignment.

File Name: Supplementary Data 2
Description: Gene expression of maturation-associated genes along zebrafish discretized maturation trajectory.

File Name: Supplementary Data 3
Description: Output of overrepresentation analysis of gene-modules based on msigdb databases.

File Name: Supplementary Data 4
Description: DTW distance and similarity for all transcription factor – target gene pairs.

File Name: Supplementary Data 5
Description: Output of Dunn's test of transcription factors similarities to target genes across modules.

File Name: Supplementary Data 6
Description: Pan-species signature orthologous mapping across species.

File Name: Supplementary Data 7
Description: Output of cross-correlation analysis of mouse and human scRNA-seq trajectories separately and species average.

File Name: Supplementary Data 8
Description: Overview of utilized external datasets.

File Name: Supplementary Data 9
Description: Summary of CellRanger metrics for the sequencing runs.

File Name: Supplementary Data 10
Description: Gene expression of common maturation-associated orthologous across the aggregated neutrophil stages and along the discretized maturation trajectories in zebrafish, mouse and human.

File Name: Supplementary Data 11
Description: Output of differential expression analysis using *FindMarkers* function from Seurat package comparing the cycling cell cluster (see Supplementary Data Fig. 3k) to other clusters.

File Name: Supplementary Movie 1

Description: **Recruitment of Mmp9$^+$ cells to wound.** Behavior and recruitment of neutrophil subpopulations with different *mmp9:Citrine* levels (*lysC:CFP$^+$/mmp9:Citrine$^+$ and lysC:CFP$^+$/mmp9:Citrine$^-$*) and macrophages (*mpeg:mcherry$^+$*) to a needle inflicted wound in triple transgenic *Tg(lysC:CFP-NTR)^{vi002}/Tg(BACmmp9:Citrine-CAAX)^{vi003}/Tg(mpeg1:mCherry)^{gl23}* zebrafish larvae at 3 dpf imaged from around 20 min post-injury. Time-lapse maximum projections. Z stacks were acquired every 50 s at 3 µm intervals, with a HC PL APO CS2 40x/1.10 WATER objective on a Leica SP8 confocal microscope using LAS X software, Zoom: 1.2 x. Scale bar = 25µm

File Name: Supplementary Movie 2

Description: **Migration behavior of an Mmp9$^+$ neutrophil showing projections around a cluster of transformed kita/RAS cells**. Representative time-lapse maximum projections acquired in quadruple transgenic *Et(kita:GAL4)^{hzm1}/Tg(UAS:EGFP-HRAS_G12V)^{io006}/Tg(lysC:CFP-NTR)^{vi002}/Tg(BACmmp9:Citrine-CAAX)^{vi003}* zebrafish larvae starting at 102 hpf with a 40x objective. Z stacks were acquired every 51 s at 2 µm intervals with a HC PL APO CS2 40x/1.10 WATER objective on a Leica SP8 confocal microscope using LAS X software, Zoom: 2 x. Scale bar = 25 µm

File Name: Supplementary Movie 3

Description: **Interactions of Mmp9$^+$ and Mmp9$^-$ neutrophils with transformed kita/RAS cells.** Representative time-lapse maximum projections acquired in quadruple transgenic *Et(kita:GAL4)^{hzm1}/Tg(UAS:EGFP-HRAS_G12V)^{io006}/Tg(lysC:CFP-NTR)^{vi002}/Tg(BACmmp9:Citrine-CAAX)^{vi003}* zebrafish larvae starting at 78 hpf. Z stacks were acquired every 3 min 39s at 3 µm intervals with a HC PL APO CS2 40x/1.10 WATER objective on a Leica SP8 confocal microscope using LAS X software, Zoom: 1.7 x. Scale bar = 50µm
