# Supplementary material for: Comparative transcriptomics coupled to developmental grading via transgenic zebrafish reporter strains identifies conserved features in neutrophil maturation
Source: Nat Commun. 2024 Feb 27;15:1792. doi: 10.1038/s41467-024-45802-1 (PMC10899643; doi:10.1038/s41467-024-45802-1)
Supplement: Supplementary file 8 — Reporting Summary [file 41467_2024_45802_MOESM8_ESM.pdf]

nature portfolio

Corresponding author(s): Stefanie Kirchberger
Florian Halbritter
Martin Distel

Last updated by author(s): 2024/01/10

Reporting Summary

Nature Portfolio wishes to improve the reproducibility of the work that we publish. This form provides structure for consistency and transparency in reporting. For further information on Nature Portfolio policies, see our Editorial Policies and the Editorial Policy Checklist.

Statistics

For all statistical analyses, confirm that the following items are present in the figure legend, table legend, main text, or Methods section.

n/a	Confirmed	
☐	☒	The exact sample size (*n*) for each experimental group/condition, given as a discrete number and unit of measurement
☐	☒	A statement on whether measurements were taken from distinct samples or whether the same sample was measured repeatedly
☐	☒	The statistical test(s) used AND whether they are one- or two-sided<br>*Only common tests should be described solely by name; describe more complex techniques in the Methods section.*
☐	☒	A description of all covariates tested
☐	☒	A description of any assumptions or corrections, such as tests of normality and adjustment for multiple comparisons
☐	☒	A full description of the statistical parameters including central tendency (e.g. means) or other basic estimates (e.g. regression coefficient) AND variation (e.g. standard deviation) or associated estimates of uncertainty (e.g. confidence intervals)
☐	☒	For null hypothesis testing, the test statistic (e.g. *F*, *t*, *r*) with confidence intervals, effect sizes, degrees of freedom and *P* value noted<br>*Give P values as exact values whenever suitable.*
☒	☐	For Bayesian analysis, information on the choice of priors and Markov chain Monte Carlo settings
☒	☐	For hierarchical and complex designs, identification of the appropriate level for tests and full reporting of outcomes
☐	☒	Estimates of effect sizes (e.g. Cohen's *d*, Pearson's *r*), indicating how they were calculated

Our web collection on statistics for biologists contains articles on many of the points above.

Software and code

Policy information about availability of computer code

Data collection	Flow cytometry: BD FACSDiva Software v9.0<br>IMC: CyTOF Software v7<br>Confocal microscopy: Leica LASX 3.7.0.20979<br>Homology mapping across species was performed using function getLDS() from biomaRt package v 2.46.3. Datasets were locally downloaded from GEO or other sources and loaded into R v 4.0.3.
Data analysis	FlowJo_v10.8.1<br>GraphPad Prism Version 8.3.0<br>ImageJ Fiji<br>QuPath-0.3.2<br>Photoshop CS6 (Adobe)<br>CellRanger v3.1.0 software (10x Genomics)<br>All bioinformatics analysis was run in R v 4.0.3. We mainly used Seurat v 4.0.2 as an infrastructure for to handle and analyze scRNA-seq data.<br><br>Computer code used for the data analysis in this paper can be accessed on our GitHub page https://github.com/cancerbits/Kirchberger_Shoeb2024_neut and via Zenodo https://doi.org/10.5281/zenodo.10475407.

For manuscripts utilizing custom algorithms or software that are central to the research but not yet described in published literature, software must be made available to editors and reviewers. We strongly encourage code deposition in a community repository (e.g. GitHub). See the Nature Portfolio guidelines for submitting code & software for further information.

Data

Policy information about availability of data

All manuscripts must include a data availability statement. This statement should provide the following information, where applicable:

- Accession codes, unique identifiers, or web links for publicly available datasets
- A description of any restrictions on data availability
- For clinical datasets or third party data, please ensure that the statement adheres to our policy

Single-cell RNA sequencing data was deposited in the Gene Expression Omnibus (GEO) and can be accessed under GSE252788. The two samples generated are accessible under:
GSM8007849 MF317_A1_GEX_zebrafish_multiseq
GSM8007850 MF317_A2_GEX_zebrafish_multiseq

We made use of the following publicly available datasets in our study:

- Single-cell RNA-seq: E-GEOD-100911, E-MTAB-5530, GSE137539, GSE165276, GSE149938, GSE142754, Myeloid cells from the fetal immune atlas, Tabula Sapiens - Immune, syn16816566
- Bulk RNA-seq: GSE79044, GSE109467, GSE172184 (neuroblastoma data provided by the authors) , GSE153263 , GSE151682 , GSE175880
- Others: neutrotime model, AnimalTFDB3.0-zebrfish, xCell signatures Additional file 5, additional metadata of GSE109467 was kindly provided by the authors.

Research involving human participants, their data, or biological material

Policy information about studies with human participants or human data. See also policy information about sex, gender (identity/presentation), and sexual orientation and race, ethnicity and racism.

Reporting on sex and gender	Bone marrow samples from patients (male and female children) with metastatic and localized neuroblastoma and ganglioneuroma have been included . There was no selection done based on sex. Neuroblastoma is slightly more abundant in male than in female children (1:1.2), but no differences in clinical outcome has been reported. Sex was considered as co-variate in the bioinformatics analysis, but was found insignificant. Sex was not considered as a co-variable in the statistical analysis of imaging data, since the number of samples did not allow for a statistically meaningful interpretation. Gender was not assessed, since the patient cohort is comprised of children aged 0-18 and the median age at diagnosis is 2 years.
Reporting on race, ethnicity, or other socially relevant groupings	Race, ethnicity or other socially relevant groupings were not assessed.
Population characteristics	In this study bone marrow aspirates from children and adolescents (age at diagnosis 0-18 years) with neuroblastoma or ganglioneuroma were analysed. The cohort used for imaging contained 21 patient samples.
Recruitment	Patients with high-risk metastatic (stage M) neuroblastoma were enrolled in the SIOPEN/HR-NBL-1 trial (NCT01704716) according to the trial's inclusion and exclusion criteria. Samples were available as left over material from diagnostic procedures and selected for this study based on their availability in the CCRI Biobank.
Ethics oversight	Ethics committee of the Medical University of Vienna, Austria

Note that full information on the approval of the study protocol must also be provided in the manuscript.

Field-specific reporting

Please select the one below that is the best fit for your research. If you are not sure, read the appropriate sections before making your selection.

☒ Life sciences ☐ Behavioural & social sciences ☐ Ecological, evolutionary & environmental sciences

For a reference copy of the document with all sections, see nature.com/documents/nr-reporting-summary-flat.pdf

Life sciences study design

All studies must disclose on these points even when the disclosure is negative.

Sample size	We did not use a statistical method to predetermine sample size. Sample size was determined as a result of previous experiments.
Data exclusions	We did not exclude data from analysis
Replication	The number of replicates for each experiment is given in the figure legends. All attempts of reproduction were successful. Experiments showed variability due to natural differences between zebrafish clutches.

Randomization	We did not perform randomization of study participants as it was not relevant for our study. Samples were available from diagnostic procedures. There were no different treatments of patients assessed.<br><br>Zebrafish experiments:<br>Fertilized eggs for standard or Morpholino injection were randomly picked from the same clutches.
Blinding	To avoid bias when assessing neutrophil morphology in Neuroblastoma patient bone marrow samples, diagnostic information (localized control or metastatic tumor) was single-blinded. Morphology assessment was done in two lots each containing samples of both diagnoses (1st lot: controls n = 4 and metastatic n = 9; 2nd lot: controls n = 5 and metastatic n = 3) . Morphology of samples was first linked to the patient number and later unblinded for each lot. Similarly, cytospin slides for assessing zebrafish neutrophil morphology were masked (single-blinded).<br><br>For other experiments (e.g. flow cytometry of standard-morpholino or Cebpb- morpholino treated larvae or Cebpb mutant analysis) no blinding was used due to technical reasons and limited staff availability.

Behavioural & social sciences study design

All studies must disclose on these points even when the disclosure is negative.

Study description	*Briefly describe the study type including whether data are quantitative, qualitative, or mixed-methods (e.g. qualitative cross-sectional, quantitative experimental, mixed-methods case study).*
Research sample	*State the research sample (e.g. Harvard university undergraduates, villagers in rural India) and provide relevant demographic information (e.g. age, sex) and indicate whether the sample is representative. Provide a rationale for the study sample chosen. For studies involving existing datasets, please describe the dataset and source.*
Sampling strategy	*Describe the sampling procedure (e.g. random, snowball, stratified, convenience). Describe the statistical methods that were used to predetermine sample size OR if no sample-size calculation was performed, describe how sample sizes were chosen and provide a rationale for why these sample sizes are sufficient. For qualitative data, please indicate whether data saturation was considered, and what criteria were used to decide that no further sampling was needed.*
Data collection	*Provide details about the data collection procedure, including the instruments or devices used to record the data (e.g. pen and paper, computer, eye tracker, video or audio equipment) whether anyone was present besides the participant(s) and the researcher, and whether the researcher was blind to experimental condition and/or the study hypothesis during data collection.*
Timing	*Indicate the start and stop dates of data collection. If there is a gap between collection periods, state the dates for each sample cohort.*
Data exclusions	*If no data were excluded from the analyses, state so OR if data were excluded, provide the exact number of exclusions and the rationale behind them, indicating whether exclusion criteria were pre-established.*
Non-participation	*State how many participants dropped out/declined participation and the reason(s) given OR provide response rate OR state that no participants dropped out/declined participation.*
Randomization	*If participants were not allocated into experimental groups, state so OR describe how participants were allocated to groups, and if allocation was not random, describe how covariates were controlled.*

Ecological, evolutionary & environmental sciences study design

All studies must disclose on these points even when the disclosure is negative.

Study description	*Briefly describe the study. For quantitative data include treatment factors and interactions, design structure (e.g. factorial, nested, hierarchical), nature and number of experimental units and replicates.*
Research sample	*Describe the research sample (e.g. a group of tagged Passer domesticus, all Stenocereus thurberi within Organ Pipe Cactus National Monument), and provide a rationale for the sample choice. When relevant, describe the organism taxa, source, sex, age range and any manipulations. State what population the sample is meant to represent when applicable. For studies involving existing datasets, describe the data and its source.*
Sampling strategy	*Note the sampling procedure. Describe the statistical methods that were used to predetermine sample size OR if no sample-size calculation was performed, describe how sample sizes were chosen and provide a rationale for why these sample sizes are sufficient.*
Data collection	*Describe the data collection procedure, including who recorded the data and how.*
Timing and spatial scale	*Indicate the start and stop dates of data collection, noting the frequency and periodicity of sampling and providing a rationale for these choices. If there is a gap between collection periods, state the dates for each sample cohort. Specify the spatial scale from which the data are taken*
Data exclusions	*If no data were excluded from the analyses, state so OR if data were excluded, describe the exclusions and the rationale behind them,*

Data exclusions	*indicating whether exclusion criteria were pre-established.*
Reproducibility	*Describe the measures taken to verify the reproducibility of experimental findings. For each experiment, note whether any attempts to repeat the experiment failed OR state that all attempts to repeat the experiment were successful.*
Randomization	*Describe how samples/organisms/participants were allocated into groups. If allocation was not random, describe how covariates were controlled. If this is not relevant to your study, explain why.*
Blinding	*Describe the extent of blinding used during data acquisition and analysis. If blinding was not possible, describe why OR explain why blinding was not relevant to your study.*

Did the study involve field work? ☐ Yes ☒ No

Reporting for specific materials, systems and methods

We require information from authors about some types of materials, experimental systems and methods used in many studies. Here, indicate whether each material, system or method listed is relevant to your study. If you are not sure if a list item applies to your research, read the appropriate section before selecting a response.

Materials & experimental systems

n/a	Involved in the study	
☐	☒	Antibodies
☒	☐	Eukaryotic cell lines
☒	☐	Palaeontology and archaeology
☐	☒	Animals and other organisms
☒	☐	Clinical data
☒	☐	Dual use research of concern
☒	☐	Plants

Methods

n/a	Involved in the study	
☒	☐	ChIP-seq
☐	☒	Flow cytometry
☒	☐	MRI-based neuroimaging

Antibodies

Antibodies used	Anti-human CD15; Supplier name: BioLegend; Catalog number: 301902; Clone name: HI98, Lot number: B265372
Validation	CD15 antibody was previously used by us (Lazic et al. doi: 10.3390/cancers13174311) and validated by the manufacturer (https://www.biolegend.com/en-us/products/purified-anti-human-cd15-ssea-1-antibody-714)

Animals and other research organisms

Policy information about studies involving animals; ARRIVE guidelines recommended for reporting animal research, and Sex and Gender in Research

Laboratory animals	Danio rerio, AB* or SAT background, larval stages and adult 3 months. The following transgenic lines were used Tg(lysC:CFP-NTR)vi002 ,Tg(lysC:dsRed)nz50Tg, Tg(mpeg1:mCherry)gl23, Et(kita:GAL4)hzm1, Tg(UAS:EGFP-HRAS_G12V)io006, Tg(HRAS_G12V:UAS:CFP)vi004, Tg(BACmmp9:Citrine-CAAX)vi003. Transgenic lines were on a AB* (Tg(lysC:CFP-NTR)vi002 ,Tg(lysC:dsRed)nz50Tg, Tg(mpeg1:mCherry)gl23, Et(kita:GAL4)hzm1, Tg(UAS:EGFP-HRAS_G12V)io006, Tg(HRAS_G12V:UAS:CFP)vi004) or AB*x SAT mixed background (Tg(BACmmp9:Citrine-CAAX)vi003) .
Wild animals	No wild animals were used in this study.
Reporting on sex	scRNAseq analysis was performed on two male zebrafish. Whether sex differences influence the neutrophil transcriptome was not assessed.
Field-collected samples	No field-collected samples were used in this study.
Ethics oversight	Zebrafish wer kept under standard conditions under licenses GZ:565304/2014/6 and GZ:534619/2014/4 according to the guidelines of the local Austrian authorities (Vienna Magistrat MA58).

Note that full information on the approval of the study protocol must also be provided in the manuscript.

Plants

Seed stocks

Report on the source of all seed stocks or other plant material used. If applicable, state the seed stock centre and catalogue number. If plant specimens were collected from the field, describe the collection location, date and sampling procedures.

Novel plant genotypes

Describe the methods by which all novel plant genotypes were produced. This includes those generated by transgenic approaches, gene editing, chemical/radiation-based mutagenesis and hybridization. For transgenic lines, describe the transformation method, the number of independent lines analyzed and the generation upon which experiments were performed. For gene-edited lines, describe the editor used, the endogenous sequence targeted for editing, the targeting guide RNA sequence (if applicable) and how the editor was applied.

Authentication

Describe any authentication procedures for each seed stock used or novel genotype generated. Describe any experiments used to assess the effect of a mutation and, where applicable, how potential secondary effects (e.g. second site T-DNA insertions, mosiacism, off-target gene editing) were examined.

Flow Cytometry

Plots

Confirm that:

☒ The axis labels state the marker and fluorochrome used (e.g. CD4-FITC).

☒ The axis scales are clearly visible. Include numbers along axes only for bottom left plot of group (a 'group' is an analysis of identical markers).

☒ All plots are contour plots with outliers or pseudocolor plots.

☒ A numerical value for number of cells or percentage (with statistics) is provided.

Methodology

Sample preparation

Adult zebrafish kidneys, spleen or blood cells were isolated by triturating. Larval zebrafish cells were isolated by Liberase Blenzyme digestion as specified in Material and Methods.

Instrument

LSRFortessa cytometer (Becton-Dickinson) and FACSAria Fusion for sorting (BD).

Software

BD FACSDiva™ 9.0 Software for data collection; FloJo_v10.8.1 for data analysis.

Cell population abundance

Describe the abundance of the relevant cell populations within post-sort fractions, providing details on the purity of the samples and how it was determined.

Gating strategy

The gating strategy for FACS sorting of cells for further scRNA processing was as follows: FSC/SSC gate, SSC-W/SSC to exclude aggregates, live gate (7AAD negative); WKM cells were sorted on live gate. To set the gate for Mmp9 NO cells, wildtype (Mmp9:Citrine negative) were used. Mmp9INT and HI gates were set to sort approximately equal numbers of cells.
Gating strategy for flow cytometry of lysC:CFP, mpeg:mCherry and mmp9:Citrine cells from transgenic fish larvae is provided in Fig. 1c and Supplementary figure 2a.

☒ Tick this box to confirm that a figure exemplifying the gating strategy is provided in the Supplementary Information.
